# Ericoid mycorrhizal fungus enhances microcutting rooting of *Rhododendron fortunei* and subsequent growth

**DOI:** 10.1038/s41438-020-00361-6

**Published:** 2020-09-01

**Authors:** Xiangying Wei, Jianjun Chen, Chunying Zhang, Hong Liu, Xiuxia Zheng, Jingli Mu

**Affiliations:** 1 Institute of Oceanography, Minjiang University, 350108 Fuzhou, Fujian Province China; 2 Department of Environmental Horticulture and Mid-Florida Research and Education Center, University of Florida, IFAS, Apopka, FL 32703 USA; 3 Shanghai Engineering Research Center of Sustainable Plant Innovation, Shanghai Botanical Garden, 200231 Shanghai, China; 4 State Key Laboratory of Ecological Pest Control for Fujian and Taiwan Crops, Fujian Agriculture and Forestry University, 350002 Fuzhou, Fujian Province China

**Keywords:** Arbuscular mycorrhiza, Plant physiology

## Abstract

Adventitious root (AR) formation is a unique feature of plant reproduction and plays a vital role in crop production as many horticultural and forestry plants are propagated through cuttings. A growing number of reports have shown that microbes, particularly mycorrhizal fungi are able to promote AR formation, but the underlying mechanisms remain largely unclear. This study established an in vitro culture system and investigated AR formation in microcuttings of *Rhododendron fortunei* Lindl. inoculated with *Oidiodendron maius* Barron Om19, an ericoid mycorrhizal fungus strain. Hormones and precursors involved in the biosynthesis of indole-3-acetic acid (IAA) in Om19 mycelium were analyzed. Om19 was able to produce a large quantity of tryptophan (Trp) and also indole-3-pyruvate (IPA) and IAA, indicating that IAA biosynthesis in Om19 could be through a Trp-dependent pathway. After inoculation of Om19, ARs were quickly formed in microcuttings. Symbiosis related genes were activated in ARs, and Om19 effectively colonized the roots. *YUC3*, a key gene in plant biosynthesis of IAA and genes involved in nitrogen (N) uptake and metabolism, phosphorus (P) uptake were highly upregulated. Plants absorbed significantly greater quantity of mineral nutrients, and their growth was substantially enhanced compared to the control plants without Om19 inoculation. A working model for Om19 enhanced AR formation was proposed. The rapid formation of ARs in cuttings could be due in part to the induction of IAA biosynthesized by Om19 and also attributed to Trp catalyzed biosynthesis of IAA in plants. AR formation, in turn, provided Om19 preferred sites for colonization. Our study suggested that in addition to promoting AR formation, Om19 could potentially be used as a new biofertilizer for enhancing production of ericaceous plants, such as blueberry, cranberry, and rhododendron.

## Introduction

Adventitious roots (ARs) are referred to as those formed post-embryonically from cells of nonrooted tissues of plants^1^. AR formation is an important component of root biology, representing a unique characteristic of reproduction and a plant’s adaptability to adverse conditions. ARs are also economically important as propagation through cuttings is central to many horticultural and forest crops^2^. Many popular ornamental crops are vegetatively propagated due to the formation ARs from various cuttings^3^, a process essential to provide several billion rooted ornamental plants to the European^4^ and the United States (U.S.)^5^ markets each year. Moreover, the formation of ARs permits clonal propagation of desirable genotypes and sports for commercial production or rapid fixation of breeding lines for crop improvement^6^.

The formation of ARs is a complex process, which can be affected by multiple factors including genetics, age, endogenous hormones, and nutritional status of plants or plant organs to be used for cutting, as well as rooting environments, such as temperature, light, relative humidity, soil or substrate properties, and soil microbes^5,6^. Thus far, how plant endogenous clues interact with environmental factors in inducing AR formation is still poorly understood even though significant progress in molecular basis on AR formation has been made recently^4,7^. Natural auxin, indole-3-acetic acid (IAA) plays fundamental roles in AR formation^8^. In *Arabidopsis thaliana* (L.) Heynh., IAA is primarily biosynthesized via a two-step pathway: Through the action of the tryptophan aminotransferase of *Arabidopsis* (TAA) family of aminotransferases, tryptophan (Trp) is converted to indole-3-pyruvate (IPA); and then IPA is converted to IAA by the YUCCA (*YUC*) family of flavin monooxygenases^9–11^. The current consensus is that the two-step or IPA pathway is essential for biosynthesis of IAA in plants, and genes in the *YUC* family catalyze a rate-limiting step in the IPA pathway^12–15^. To enhance AR formation, many synthetic auxins, including naphthaleneacetic acid (NAA) and indole-3-butyric acid (IBA) are commonly applied exogenously in the propagation of horticultural and forest plants.

Since the 1970s, increasing reports have shown that soil microbes, particularly mycorrhizal fungi play important roles in enhancing AR formation^16–21^. There are four main types of mycorrhizal associations, typically known as arbuscular mycorrhiza (AM), ectomycorrhiza (EcM), ericoid mycorrhiza (ErM), and orchidaceous mycorrhiza (OrM)^22^. Arbuscular mycorrhizal fungi commonly form a mutualistic relationship with the roots of a wide range of crops. The benefits of AMs are most pronounced when fungal colonization occurs at the earliest stage of plant growth^23,24^. In plant propagation by cuttings, it is recommended that maximum benefits from AM fungal symbiosis can be achieved if inoculum is present during AR formation. However, mechanisms underlying AM fungus-mediated AR formation are largely uncertain^4,17,25^.

Fungi involved in the formation of ErM include Ascomycetes in the class Leotiomycetes and some Basidiomycetes in the Serendipitaceae^26^. The most important ascomycetous ErM fungi belong to the Hyaloscypha hepaticicola aggregate, formerly known as the Hymenoscyphus/Rhizoscyphus ericae aggregate^26^. The basidiomycetous ErM comprise non-sebacinoid fungi^27^ and sebacinoid fungi from the Serendipitaceae^28^. *Oidiodendron maius* Barron, a member of Leotiomycetes^28^ can form ErM with plants in the family Ericaceae and has also been isolated from other plant roots, as well as decayed organic materials, peat, and acidic soil^29^. *Rhododendron fortunei* Lindl., a member of the family Ericaceae, is one of the most popular ornamental flowering plants, particularly in China. Besides its ornamental value, *R. fortunei* is also an important industrial crop^30^. Other economically important crops in the family Ericaceae include bilberry (*Vaccinium myrtillus* L.), blueberry (*Vaccinium* sect. *Cyanococcus* Rydb. spp.), cranberry (*Vaccinium* subg. *Oxycoccus* (Hill) A. Gray spp.), and huckleberry (*Vaccinium parvifolium* sm.). These plants are calcifuges, growing naturally in acidic soil with low fertility^29^. Stem cuttings used to be a main method of propagating this group of plants. Górecka^31^ reported that rooting of cuttings derived from ericaceous plants was difficult as low rooting percentages occurred when cuttings were rooted outside an optimal time. In addition, even after rooting, rooted cuttings had poor survival rates after transplantation. Recently, propagation of ericaceous plants, particularly blueberry and rhododendron has been shifted to micropropagation, primarily shoot culture. An interesting phenomenon in shoot culture of blueberry and rhododendron is that in vitro rooting of microcuttings produced from axillary shoots is more difficult than ex vitro rooting in commercial substrates^30,32,33^. This could be due to the occurrence of mycorrhizal fungi in commercial substrates, which promote rooting of ericaceous plants. ErR fungi have been found to colonize ericaceous plants in nursery production^34^. Rooting of blueberry cuttings inoculated with ErM fungi was more successful, and plant growth was enhanced^21,35^. A recent report showed that inoculation of ErM fungi significantly increased root and shoot biomass of bilberry^36^. Our previous study showed that seedlings of *R. fortunei* inoculated with an ErM fungus (*O. maius* strain Om19) produced abundant roots^37,38^. Furthermore, we observed that some senesced leaves after dropping from in vitro cultured *R. fortunei* onto a peat-based substrate inoculated with Om19 were able to produce ARs, but no ARs appeared in the fallen leaves on the same substrate devoid of Om19. We hypothesized that ErM fungi could play important roles in promoting rooting and subsequent growth of ericaceous plants. However, similar to AM-mediated rooting, mechanisms behind the ErM-enhanced rooting remain primarily unknown.

The objective of this study was to explore biochemical and molecular bases underlying the ErM fungus-mediated rooting of ericaceous plants using *R. fortunei* as a model. We developed an in vitro culture system for studying symbiosis of *R. fortunei* with Om19, analyzed hormone production in Om19, and determined relevant gene expression in plants. A working model to illustrate molecular mechanisms underlying the enhanced rooting of ericaceous plants was proposed.

## Results

### AR formation and symbiosis establishment

**Fig. 1:**
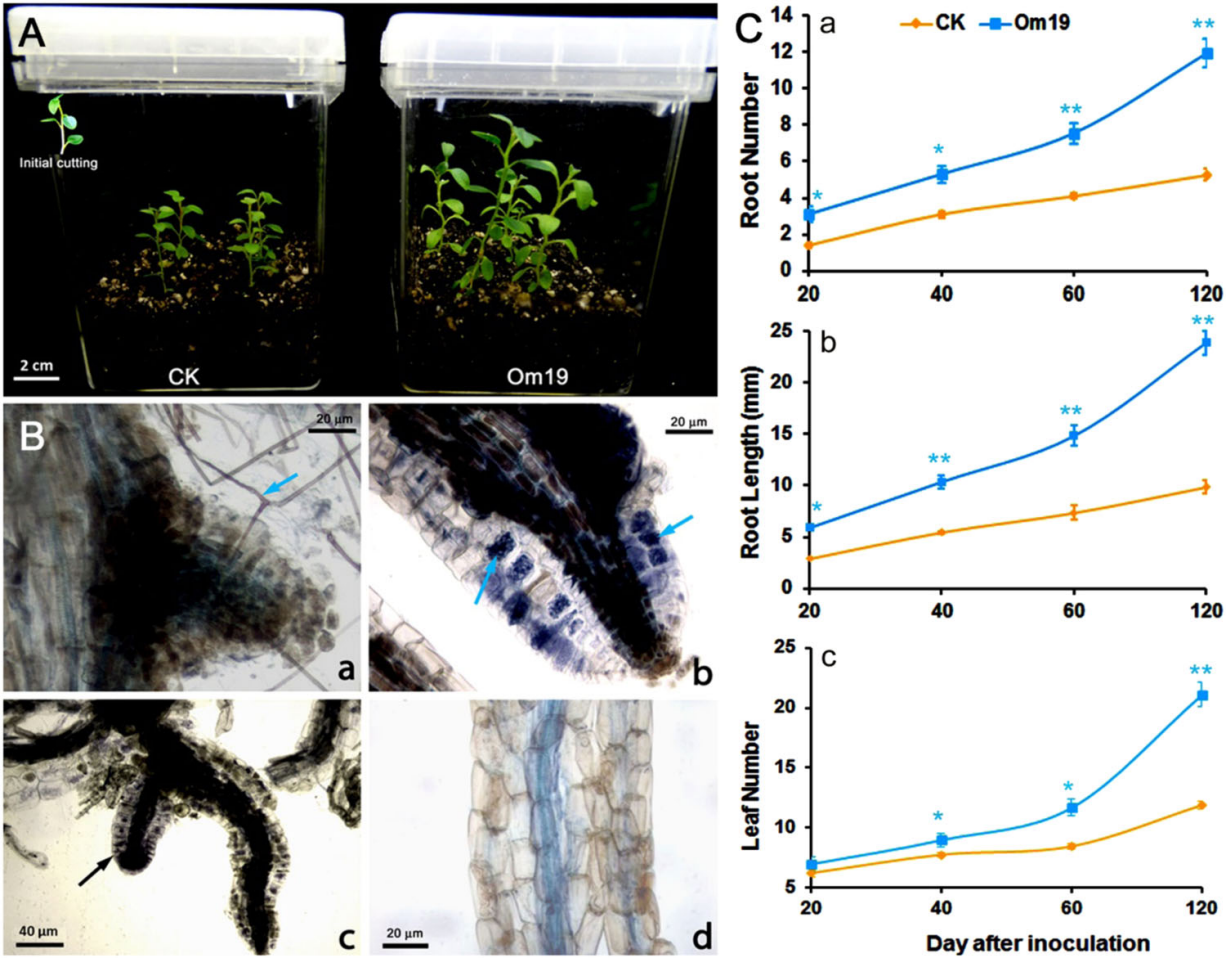
In vitro culture system for studying adventitious root formation, symbiosis, and plant growth. **a** Microcuttings (the insertion on the upper left corner) derived from in vitro cultured *R. fortunei* grown in a sterilized peat-based substrate without (CK) and with inoculation of Om19 (Om19). **b** AR formation and Om19 colonization: Mycelium (sky blue arrow) of Om19 appeared on the surface of an AR formed from the base of a cutting in one weeks (**a**), mycelium coils (sky blue arrow) occurred in rhizodermal and cortex cells of ARs in two-three weeks (**b**), rhizodermal and cortex cells of lateral roots (black arrow) were colonized by Om19 with dense coin in four weeks (**c**), and no mycelium on root surface or rhizodermal cells of ARs formed from cuttings without Om19 inoculation (**d**). **c** The growth of ARs and shoot leaf numbers: AR number (**a**), average root length (**b**), and leaf numbers (**c**) of microcuttings produced during a 120-day growth period in the sterilized peat-based substrate uninoculated (CK) and inoculated (Om19) with Om19. Bars represent standard errors (*n* = 5) where (*) and (**) indicate significant differences in a given parameter between CK and Om19 treatments in the sampling date based on Tukey’s HSD test at *P* < 0.05 and *P* < 0.01 levels, respectively

The in vitro culture system developed for studying AR formation from microcuttings of *R. fortunei* and symbiosis between Om19 and ARs was illustrated in Fig. 1a. Cuttings (the insertion of Fig. 1a) derived from axillary shoots of in vitro cultured stems were stuck in a sterilized peat-based substrate and inoculated with Om19. ARs were produced in a week, while those stuck in the substrate without Om19 produced ARs in about two weeks. Light microscopy examination showed that mycelium presented on the surface of ARs formed from the base of cuttings in a week (Fig. 1b-a), mycelium coils occurred in the rhizodermal and cortex cells of ARs in two-three weeks (Fig. 1b-b). Almost all examined ARs and lateral roots were colonized by Om19, and root rhizodermal and cortex cells of the roots were nearly filled with mycelium coils in four weeks (Fig. 1b-c). However, no mycelium was observed on root surface or rhizodermal cells of ARs formed from cuttings rooted in the control substrate, i.e., without inoculation of Om19 (Fig. 1b-d). Morphological parameters of ARs, mainly the number of roots and root length and also leaf numbers of rooted cuttings were recorded after 20, 40, 60, and 120 days of sticking (Fig. 1c). Microcuttings inoculated with Om19 had significantly more roots (Fig. 1c-a) and longer average root length (Fig. 1c-b) than those uninoculated with Om19 from day 20 to 120. There was no significant difference in leaf numbers on day 20 between inoculated and uninoculated cuttings. However, significantly more leaves were observed in microcuttings inoculated with Om19 than those uninoculated with Om19 from day 40 to 120 (Fig. 1c-c).

### Plant growth during in vitro culture

**Fig. 2:**
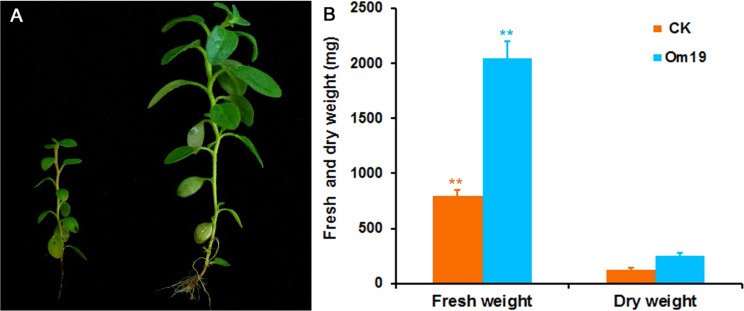
Growth of *Rhododendron fortunei* microcuttings in a sterilized peat-based substrate. **a** Microcuttings rooted in the peat-based substrate without (CK) and with inoculation of Om19 (Om19) after 120 days of growth. **b** Fresh and dry weights of 40 rooted cuttings grown in the peat-based substrate without (CK) and with Om19 inoculation. Bars represent standard errors (*n* = 3) where (**) indicates significant differences in a given parameter between microcuttings uninoculated (CK) and inoculated (Om19) with Om19 based on Tukey’s HSD test at *P* < 0.01 levels

Microcuttings grown in Om19 inoculated substrate were morphologically different from those grown in the control substrate. They were taller, leaves were larger, and stem diameter was greater (Fig. 2a). At the end of in vitro evaluation experiment, i.e., 120 days after rooting, the fresh weight of 40 plants inoculated with Om19 (plants from 10 vessels, 4 per vessel) was 2,051.3 mg compared to 796.7 mg for the control plants (Fig. 2b). The dry weight of the 40 plants inoculated with Om19 was 254.4 mg compared to 128.5 mg for the control plants. Both fresh and dry weight of plants inoculated with Om19 were significantly greater than those uninoculated with Om19.

Tissue analysis of entire plants harvested after 120 days of growth showed that there were no significant differences in concentrations of macronutrients except P, K, and Mg which were significantly higher in plants colonized by Om19 than those of control plants (Table 1). Due to the difference in biomass (Fig. 2b), Om19 colonized plants took up significantly greater quantity of macronutrients from the substrate than the control plants.

**Table 1:** Macronutrient concentrations and contents of microcuttings of *Rhododendron fortunei* grown in a sterilized peat-based substrate uninoculated and inoculated with Om19

Treatments	Conc./content	N	P	K	Mg	Ca	S
Uninoculated	Conc. (%)	1.4	0.6	1.6	0.3	0.5	0.2
Inoculated	Conc. (%)	1.6	1.2*	2.7*	0.6*	0.6	0.3
Uninoculated	Total content (mg)	1.82	0.71	2.03	0.39	0.66	0.27
Inoculated	Total content (mg)	4.12*	2.93**	6.92*	1.40**	1.63*	0.81*

Total contents refer to a total amount (mg) of individual nutrients in 40 plants

Data were means where (*) and (**) indicate significant differences in concentration or total content between plants uninoculated and inoculated with Om19 at *P* < 0.05 and *P* < 0.01 levels, respectively, based on Tukey’s HSD test (*n* = 3)

### Hormones and precursors of IAA produced by Om19

**Fig. 3:**
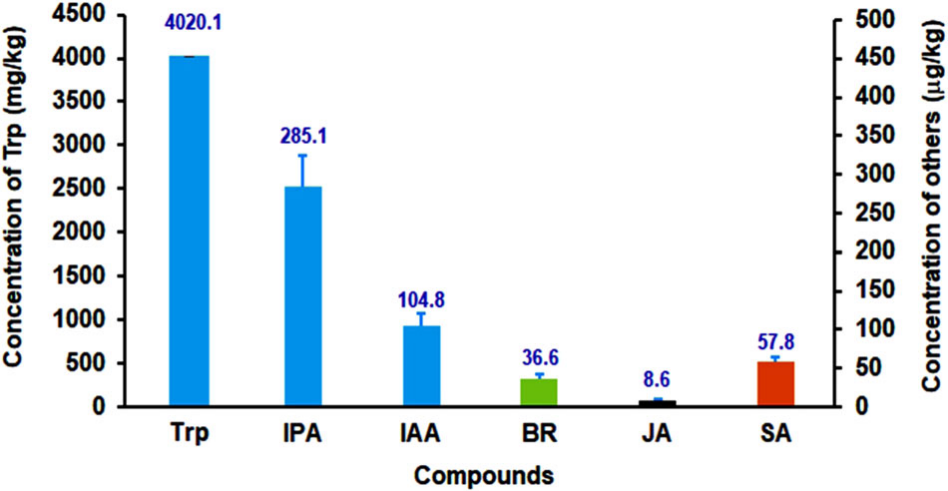
Hormones and tryptophan (Trp) synthesized by Om19. Concentrations of Trp, indole-3-pyruvate (IPA), indole-3-acetic acid (IAA), brassinolide (BR), jasmonic acid (JA), and salicylic acid (SA) detected in mycelium of Om19. Bars represent standard errors (*n* = 3)

Mycelium of Om19 was analyzed for hormones and precursors involved in IAA biosynthesis. Results showed that Trp was extremely higher with a mean concentration of 4,020.1 mg kg^−1^ (Fig. 3). Other compounds detected were IPA at 285.1 μg kg^−1^, IAA at 104.8 μg kg^−1^, brassinolide (BR) 36.6 μg kg^−1^, jasmonic acid (JA) 8.6 μg kg^−1^, and salicylic acid (SA) 57.8 μg kg^−1^. Whereas, abscisic acid, gibberellins (GA_3_), strigolactone, trans-zeatin, and trans-zeatin 6-(4-hydroxy-3-methylbut-2-enylamino) purine were not detected in Om19 mycelium.

### RT-qPCR analysis of relevant gene expression

**Fig. 4:**
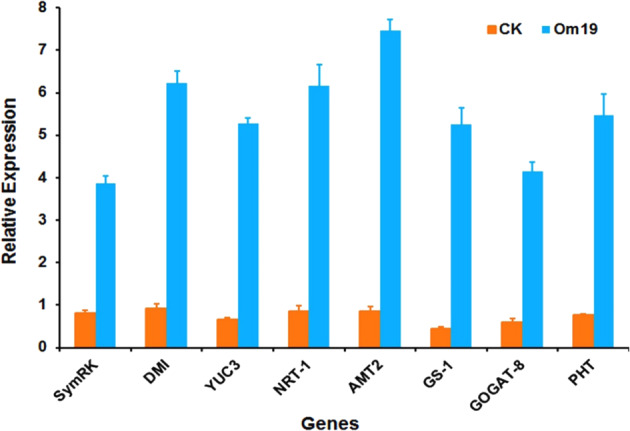
Om19 colonization resultant gene expressions in plant roots. The expression levels of eight genes involved in symbiosis (*SymrK* and *DMI*), plant auxin biosynthesis (*YUC3*), N uptake (*NRT1* and *AMT*) and metabolism (*GS-1* and *GOGAT-8*), and phosphorus (*PHT*) absorption in roots of *R. fortunei* inoculated with (Om19) were significantly higher than those uninoculated with Om19 (CK)

RT-qPCR analysis showed that *SymRK* and *DMI3*, which are involved in the symbiosis establishment between mycorrhizal fungi and plants, were highly upregulated in roots inoculated with Om19 compared to the uninoculated control plants (Fig. 4). The expression of *YUC3*, a key gene responsible for IAA biosynthesis from the Trp-dependent pathway, was particularly high in plants colonized by Om19. *NRT1* and *AMT2* which are involved in nitrate and ammonium uptake, *GS* and *GOGAT* which are directly responsible for N metabolism, and *PHT*, a phosphate transporter, were all highly upregulated in Om19-colonized roots. Overall, the expression levels of these genes in roots inoculated with Om19 were 3.7 to 10.7 times greater than those uninoculated with Om19.

### Ex vitro rooting and plant growth in greenhouse

**Fig. 5:**
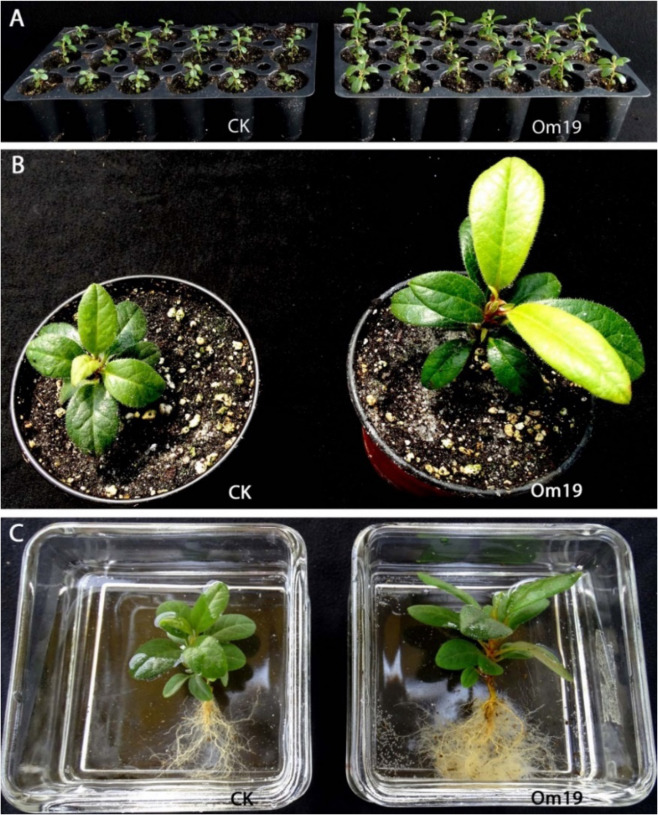
Ex vitro rooting of *Rhododendron fortunei* microcuttings uninoculated (CK) and inoculated (Om19) with Om19 in a Canadian peat-based substrate and subsequent growth in a shaded greenhouse. **a** Two months after microcuttings were rooted in 18-cell trays, (**b**) three months after being transplanted to 10-cm diameter containers, and (**c**) hair roots of plants after washing away of substrate

Microcuttings were rooted in a Canadian peat-based substrate uninoculated and inoculated with Om19 in a shaded greenhouse (Fig. 5). ARs were formed from cuttings in about a week regardless of inoculation or uninoculation with Om19, but significantly more roots numbers occurred in cuttings inoculated with Om19 after 30 days of transplanting (Table S1). After 60 days of growth in plug trays, root numbers were not recorded due to the difficulty in counting hair roots. Leaf numbers were comparable, but plant canopy height and leaf area of the largest leaf inoculated with Om19 were significantly greater than the control (Fig. 5a, Table S1). The plants were then transplanted to 10-cm diameter containers containing the same substrate and grown in the same shaded greenhouse for additional three months. Leaf numbers, canopy height, and leaf area of the largest leaf of Om19 inoculated plants were significantly greater than the control plants (Fig. 5b, Table S1). Moreover, plants inoculated with Om19 had much more abundant hair roots than the control plants (Fig. 5c).

## Discussion

Mycorrhizal fungus-mediated AR formation has been reported more than 40 years ago^16^, but underlying mechanisms remain largely unclear^17,18,38–40^. In this study, microcuttings of *R. fortunei* derived from in vitro shoot culture were stuck in a sterilized peat-based substrate inoculated with an ErM fungus Om19, AR formation, Om19 colonization, and the colonization resultant plant growth were investigated (Fig. 1). Our results showed that microcuttings inoculated with Om19 produced ARs in a week compared to two weeks from the control substrate. Such a rapid formation of ARs could be attributed to two factors: One could be the effect of IAA produced by Om19 (Fig. 3). Om19 was able to produce Trp, IPA, and IAA, suggesting that IAA biosynthesis in Om19 is likely through a Trp-dependent biosynthesis pathway, i.e., from Trp to IPA, IPA to IAA as reported by Krause (2015)^40^ in *Tricholoma vaccinum*. The synthesized IAA could promote mycelium growth, similar to the report of Krause (2015)^40^, i.e., IAA synthesized by *T. vaccinum* induced cell elongation and hyphal ramification. Meanwhile, IAA produced by Om19 might also induce microcuttings to form ARs. Mycelium of *Laccaria bicolor*, an ectomycorrhizal fungus, was able to synthesize IAA and induced root formation in poplar (*Pupulus* spp.)^41,42^. IAA produced by an ectomycorrhizal fungus *Hebeloma cylindrosporum* promoted rooting of microcuttings obtained from *Pinus pinaster* and *P. sylvestris*^43^. The ARs in turn could provide the preferred infection sites for Om19. Several in vitro studies also showed that fungi with high IAA production capacity colonized more root tips and formed mycorrhizae at a higher rate than fungi with low IAA production^44–46^. Mycorrhiza establishments stimulated abundant hair root growth in *R. fortunei*^37^ and root hair growth of trifoliate orange (*Poncirus trifoliata*)^47^. In this study, Om19 colonized not only ARs, but also lateral roots (Fig. 1b-c).

The other factor could be the action of IAA produced by plants. Plant can synthesize IAA using Trp, known as the Trp-dependent IAA biosynthetic pathway. IAA can also be synthesized by Trp-independent pathway^48^. Within the Trp-dependent pathway, four pathways have been proposed in *Arabidopsis*, namely the indole-3-pyruvic acid (IPA) pathway, the indole-3-acetaldoxime (IAO) pathway, tryptamine (TAM) pathway, and the indole-3-acetamide (IAM) pathway^49^. Thus far, it is unknown which IAA biosynthesis pathway occurs in *R. fortunei*. Our results with highly upregulated *YUC3* expression (Fig. 4) may suggest the IPA could be a pathway for IAA biosynthesis in *R. fortunei*, which could be the same pathway as IAA biosynthesis in Om19. Since a large quantity of Trp was produced by Om19, the Trp could be released to the substrate and absorbed by microcuttings or released to plant cells due to the symbiosis. Elevated Trp in microcutting might catalyze IAA biosynthesis in plants. IAA produced from both Om19 and the plant could provide a synergistic effect on AR formation as shown in Fig. 1-c.

**Fig. 6:**
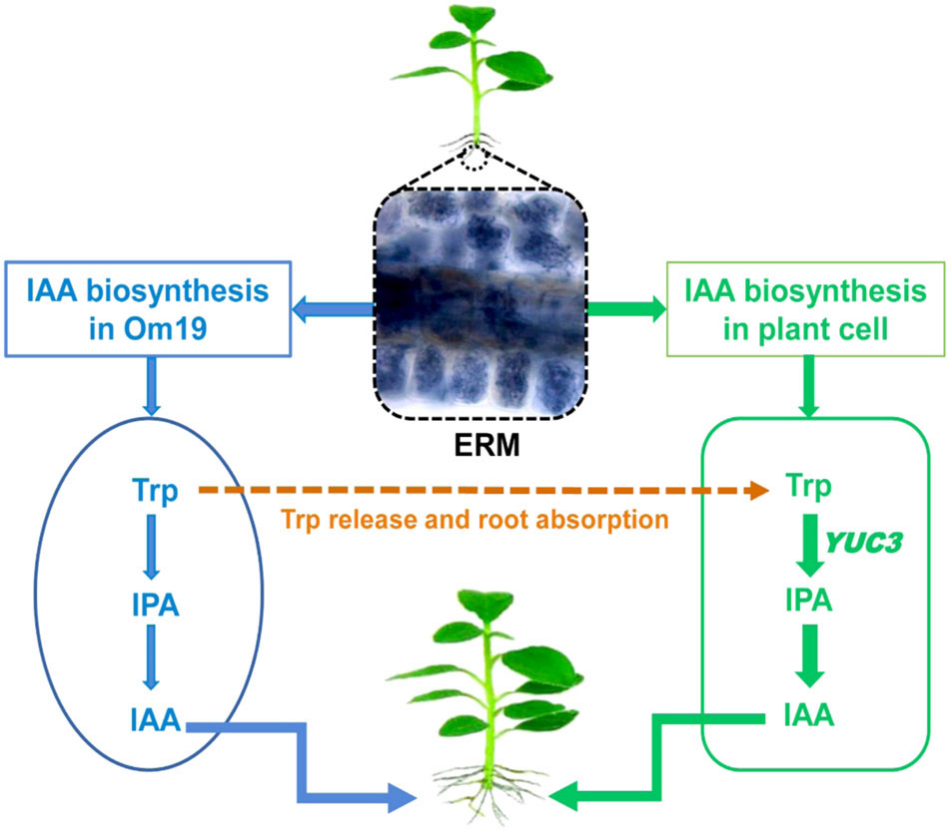
A working model for Om19-enhanced rooting of microcuttings. Microcuttings of *Rhododendron fortunei* stuck in a sterilized peat-based substrate inoculated with Om19. Om19 was able to produce a high concentration of tryptophan (Trp), which was used for synthesizing indole-3-acetic acid (IAA) through indole-3-pyruvate (IPA) to promote mycelium growth. The IAA may also induce AR formation in microcuttings. Meanwhile, the high Trp may catalyze biosynthesis of IAA in plant cells as *YUC3*, a key gene in plant biosynthesis of IAA, was highly induced. IAA produced by Om19 and plant could enhance AR formation

Taking all the results into account, we proposed a working model to illustrate the Om19-enhanced rooting and subsequent growth of microcuttings (Fig. 6). Our observations showed that Om19 became quickly established in the substrate, taking less than two days. The establishment could lead to its biosynthesis of Trp and IAA. The synthesized IAA would induce hyphal growth for establishing colonization. At the same time, the IAA might also induce AR formation of microcuttings. Furthermore, since a large quantity of Trp was produced by Om19 (Fig. 3), this could be a catalyst to IAA biosynthesis in plants. IAA produced from plant and Om19 might accelerate AR initiation, root elongation, and root growth in general (Fig. 1c), which in turn provided more root surfaces for Om19 colonization. The increased root numbers and length due to Om19 colonization enhanced plant uptake of nutrients. The increased expression of *NRT-1*, *AMT2*, and *PHT* suggested that plants effectively absorbed NO_3_^−^, NH_4_^+^, and P (Table 1). Similar to AM fungi, mycelium of Om19 took up NO_3_^−^, which was converted to arginine; through the action of arginase and urease, ammonium was released from arginine to plant roots^37^. The elevated ammonium in plants could induce *GS* and *GOGAT* activities. RT-qPCR analysis documented that *GS-1* and *GOGAT-8* expressions were significantly upregulated in Om19 colonized plants (Fig. 4). GS and GOGAT are key enzymes in the synthesis of glutamine and glutamate, which resulted in the bioavailability of organic N in plant metabolism, such as nucleic acid, amino acid, and polysaccharide biosyntheses. As a result, the growth of plants colonized by Om19 was significantly enhanced compared to those without Om19 colonization. Om19 colonized plants grew significantly larger and healthier than control ones (Fig. 2), similar to the results of Wei et al.^37,38^. On the other hand, the control plants would solely depend on the adaptation of microcuttings to the substrate and then biosynthesize IAA. As a result, AR formation in control plants was slower, and root numbers and root length were less than Om19-colonized plants.

Om19 also produces BR, JA, and SA. BRs derived from plants have been shown to positively affect the symbiosis between tomato or tobacco and an AM fungus^50^. JAs serve as a wound signal during AR formation, and they can be accumulated in plant tissues just a few minutes after wounding. It has been proposed that increased JA concentration promotes the formation of ARs in cuttings through accumulation of IAA in the base of stems and/or canalization towards AR source cells^4^. Felten et al. (2010)^51^ suggested that JA might act a molecular signal for inducing lateral roots. SA is known for its induction of systemic-acquired resistance^52^. A recent study showed that low levels of SA promoted AR formation and altered the root apical meristem architecture, whereas high levels of SA suppressed the root growth processes^53^. How BRs, JA, and SA synthesized by Om19 affect AR formation and subsequent growth of *R. fortunei* is unknown at this point, and certainly deserves further investigation.

The time to induce AR formation from inoculated and uninoculated microcuttings during ex vitro rooting was similar. An explanation is the formulated substrate with Canadian peat might have ErM mycorrhizal fungi. Gorman and Starrett (2003)^54^ evaluated commercial peat-based substrates used in the U.S. and Canada and found that majority of the peat and peat-based substrates contained ErM fungi. The ErM fungi in the substrate may behave similar to Om19, promoting AR formation. Thus, AR formation between cuttings inoculated and uninoculated with Om19 occurred at the same time. This may also explain why in vitro rooting in aseptic conditions is more difficult than ex vitro rooting of rhododendron and blueberry^30,32,33^. Several studies have shown ErM fungi colonize ericaceous plants naturally; but inoculation of ErM fungi increased colonization and improved plant growth^21,34,35^. Our greenhouse results are consistent with those observations (Fig. 5). On the other hand, a few studies indicated that ErR inoculation did not improve AR formation^39,55^ or produced variable results based on season^31^. These could be attributed to the difference in mycorrhizal fungal species and even strains^55^, as not all strains could act like Om19 and are able to produce high concentrations of Trp and IAA. Another possibility could be due to the adaptability of fungal strains to different environmental conditions. Some ErM species or strains could perform better in certain substrates or certain rooting environments^31^.

## Conclusion

The formation of ARs is a unique and basic process of root biology and of great importance in clonal propagation. For the first time, our study documents that Om19 can synthesize a large quantity of Trp, as well as IPA and IAA, indicating that IAA could be produced through a Trp-dependent pathway. Om19 also produced BR, JA, and SA. IAA produced by Om19 was required for mycelium growth, and it may also induce AR formation in microcuttings. Furthermore, Trp produced by Om19 could be used by plants to biosynthesis of IAA. IAA biosynthesized by Om19 and plants might act together to enhance AR formation. Om19 was able to establish symbiosis with roots in two weeks. The colonization induced the expression of genes involved in symbiosis, IAA biosynthesis, N uptake and metabolism, and P uptake. Plants colonized by Om19 were more effective in absorption of macronutrients, and as a result, plant growth was substantially enhanced by Om19 colonization. This is the first attempt to elucidate molecular mechanisms underlying Om19-enhanced AR formation and plant growth. Further research is warranted to verify and improve the proposed model. In addition, this study showed that Om19 could be a valuable agent to be used for improving production of ericaceous plants, such as blueberry, cranberry, and rhododendron.

## Materials and methods

### Plant material, Om19, and in vitro culture system

Microcuttings of *R. fortunei* (the insertion of Fig. 1a) were derived from three-month-old axillary shoots, approximately 2.5 cm in height with 5–6 leaves. The axillary shoots were produced from two-node explants cultured on Woody Plant Medium (PhytoTech Labs, Shawnee Mission, KS, USA) supplemented with 1.0 mg L^−1^ NAA and 4.0 mg L^−1^ zeatin or 6-(4-hydroxy-3-methylbut-2-enylamino) purine described by Wei et al. (2018)^30^. The nodes were originally produced from in vitro germinated seedlings.

To establish an in vitro culture system for symbiosis, a peat-based substrate was produced which was composed of 60% dry Klasmann peat (Geeste, Germany) and 30% dry sand by volume. The sand was washed with tap water twice, then deionized water five times, and dried. Total nitrogen (N) and organic matter contents of the Klasmann peat were 0.89% and 96.5%, respectively. The formulated peat-based substrate was moistened with a fertilizer solution in a 3:2 ratio by volume. The solution was made from the Peters Professional 20–20–20 (N-P_2_O_5_-K_2_O) General Purpose Fertilizer (Scotts Co., Marysville, OH, USA) with a N concentration at 100 mg L^−1^. After adjusting the pH to 5.2, each Magenta GA-7 culture vessel (MilliporeSigma, Burlington, MA, USA) was filled with 100 mL of the substrate and covered with the manufacturer-supplied cap. The substrate-filled culture vessels were autoclaved at 121 ^o^C for 30 min. After cooling to room temperature inside laminar flow hoods, the aforementioned microcuttings were stuck into 88 culture vessels, four cuttings per vessel.

An *O. maius* strain Om19 was initially isolated from roots of *R. fortunei*^37^, which was cultured on modified Melin-Norkans agar medium (MMN)^56^. After two weeks of culture, Om19 mycelium were taken using sterilized cork borers with a size at 5 mm diameter. The 5-mm discs were cut in half (with a surface area about 10 mm^2^) and placed to the surface of the peat-based substrate next to each cutting. A total of 44 culture vessels were inoculated with Om19. The remaining 44 were used as the control by placing the same sized disc of MMN devoid of Om19 adjacent to each cutting. Twenty of the 44 vessels per treatment were used for monitoring rooting and plant growth. This experiment was set as a completely randomized design with five replications. The remaining 24 vessels per treatment were used for monitoring symbiotic establishment between Om19 and ARs. All culture vessels were maintained in a culture room which was lighted by cool-white fluorescent lamps at a photon flux density of 50 µmol m^−2^ s^−1^ under a 16-h photoperiod. The temperature of the culture room was set at 25 ^o^C. To record rooting and plant growth, one vessel per treatment was taken from each replication after 20, 40, 60, and 120 days of sticking. Rooted cuttings were recovered by inverting the vessels in distilled water and washing roots free from substrate debris. Root numbers, mean root length, and leaf numbers of cuttings in each vessel were counted or measured after plants were blotted dry with paper towel. Means of each parameter were calculated, and all data were presented as mean ± standard error (S.E.).

### Microscopic observation of ErM established roots of cuttings

The remaining 24 vessels per treatment were used for monitoring ErM establishment beginning on the fifth day after cutting insertion. Three vessels per treatment were randomly taken, and cutting were recovered as mentioned above. When ARs were equal or greater than 1 mm, they were excised from microcuttings and fixed immediately in FAA solution (formaldehyde-acetic acid-ethanol) for 24 h and then cleared at 90 ^o^C in a 10% KOH solution for 1 h. Using 1% HCl solution, the roots were acidified and then stained with a solution known as lactophenol-trypan blue (0.05% trypan blue in lactophenol) at 90 °C for 5 min^57^. After clearing with fresh lactophenol, the roots were examined under a light microscope for ErM structures in roots^37^. Such observations were repeated every other day thereafter for a total of 19 days.

### Plant biomass and nutrient analysis

To determine biomass and tissue nutrient concentrations, another experiment was conducted. Microcuttings were stuck in 60 culture vessels, each containing 100 mL of the autoclaved peat-based substrate mentioned above, four cuttings per vessel. Cuttings in 30 culture vessels were inoculated with Om19 as described above, and the remaining 30 were the control treatment. The experiment was a randomized complete block design with three replications, i.e., each block had 10 vessels. Rooted cuttings or plants were harvested after 120 days of growth. After roots were washed and rinsed in distilled water three times, plants were immediately blotted dry on paper towels. In order to have enough tissue for nutrient analysis, plants from each block (a total of 40) were combined, fresh weight was recorded. The plants were dried at 80 ^o^C for 48 h, and dry weight was recorded. For determining tissue N contents, plants were analyzed using CNS Auto-Analyzer (VarioMAX, Elementar Americas, Mt. Laurel, NJ, USA) and other elements using PerkinElmer Optima 8399 ICP-OES (Billerica, MA, USA).

### Analysis of tryptophan and hormones in mycelium of Om19

Om19 was cultured in three flasks containing potato dextrose broth medium on a rotating shaker at 150 rpm, 25 ^o^C in total darkness for seven days. Mycelium was collected by filtration through Whatman filter paper No. 1 and frozen in liquid N. Tryptophan (Trp) was analyzed using mass spectrometry based on the method described by Krause et al. (2015)^40^.

For analysis of hormones, mycelium was extracted by acetonitrile or methanol and diluted by diethyl ether to different concentration gradients. Based on the reference of Šimura et al. (2018)^58^, abscisic acid, brassinolide, indole-3-pyruvic acid, indole-3-acetic acid, indole-3-butyric acid, 1-acetylindole, gibberellins (GA_3_), jasmonic acid, salicylic acid, strigolactone, tryptophan, trans-zeatin, and trans-zeatin 6-(4-hydroxy-3-methylbut-2-enylamino) purine were analyzed using the Thermo Scientific™ Q Exactive™ HF hybrid quadrupole-Orbitrap mass spectrometer by Sci-Tech Inovation, Inc. (Qindao, China). CSH C18 RP column (150 × 2.1 mm, particle size of 1.7 μm) was used, and the analysis conditions were set as follows: cone/desolvation gas flow at 150/1000 L h^−1^; capillary voltage at 2.1 kV for ESI(+) and 1.5 kV for ESI(–); source/desolvation temperature at 125 °C/600 °C; collision energy at 12 to 30 eV; cone voltage at 10 to 40 V; and collision argon gas flow at 0.21 mL min^−1^.

### RNA extraction

To determine if key genes involved in Om19 colonization, IAA biosynthesis, N and phosphorus (P) uptake, and N metabolism were differentially expressed between inoculated and uninoculated plants, a third plant growth experiment was conducted to produce sufficient root materials for extracting RNA. The experiment was set up in the same way as those mentioned above except that there were 300 culture vessels. Microcuttings in 150 vessels were inoculated with Om19 and labeled as Om19, and the remaining 150 vessels were used as the control without Om19 inoculation and labeled as CK. After eight weeks of inoculation, roots from cuttings were collected from each vessel by washing roots in tap water free from substrate, rinsing with sterile deionized water three times, and blotting dry with paper towel. Roots from 50 vessels per treatment were pooled, resulting in three biological samples, frozen immediately in liquid N, and stored at −80 ^o^C, respectively. Total RNA from the collected roots was extracted and RNA quality was tested as described by Wei et al.^38^.

### RT-qPRC analysis of genes related to symbiosis, IAA biosynthesis, and N and P uptake

RT-qPCR analysis was conducted to determine the expression level of *SymRK* (Unigene22160_All), *DMI3* (Unigene3610_All), *YUC3* (Unigene25509_All), *NRT1* (Unigene35804_All, nitrate transporters), *AMT* (CL4699.Contig1_All, ammonium transporters), *GS* (CL10295.Contig2_Al, glutamine synthetase), *GOGAT* (Unigene19965_All, glutamate synthase), and *PHT* (Unigene15587_All, phosphate transporter) in plant roots. These genes were based on the transcriptome data sets of our previous study^38^, which is available with an accession number SRP064996 at the NCBI Sequence Read Archive (SRA). Both *SymRK* and *DMI3* are involved in early symbiosis processes^38^. Primers specifically for *R. fortunei* were designed according to the cDNAs with Primer Premier software (version 5.0) (Supplemental Table S2). *EF1α* was used as an internal control, which was tested to be the most stably expressed gene in our previous study^37^. The PrimeScript 1st cDNA Synthesis Kit (Promega, WI, USA) was used to synthesize the first strand cDNA. RT-qPCR was performed in a 20 μL reaction mixture containing 2× SYBR (PerfeCTa SYBR Green FastMix, Low ROX) 10.0 μL (Quantabio, MD, USA), 1 μL of each corresponding primer for EF1α and the gene of interest, and 1.5 μL of cDNA template (1:5 dilution). RT-qPCR of three biological replicates per treatment was conducted using a LightCycler 480 II System for 5 s at 95 °C, 10 s at 56 °C, and 20 s at 72 °C. The relative expression levels were calculated and normalized based on the 2^−ΔΔCT^ method^59^. For each given gene, the relative expression level was presented as mean ± SE of three replicates.

### Ex vitro inoculation

Microcuttings (about 3-cm in height) derived from in vitro shoot culture were stuck in 18-cell plug trays filled with a Canadian peat-based substrate composed of 60% Canadian peat, 20% vermiculate, and 20% perlite by volume. The substrate pH was adjusted to 5.2 using a commercial dolomite. The substrate was moistened with the Peters Professional 20–20–20 General Purpose Fertilizer (The Scotts Co., Marysville, OH, USA) solution containing 100 mg L^−1^ N. Om19 was cultured on MEA for two weeks, and Om19 mycelium was collected using sterilized 5-mm cork borers. Six plugs (5-mm diameter discs) were placed into aseptic 150 conical flasks containing 80 ml of sterilized liquid MMN and cultured in a rotary shaker (150 min^−1^) at 25 °C for 10 days in total darkness. Six plugs derived from MEA medium without Om19 were also cultured in the 150 conical flasks containing 80 ml of sterilized liquid MMN as a control treatment. The culture was fragmented with a blender and inoculated using a pipette by placing 1 mL of the inoculum into each plug cell. There were three trays per treatment (with and without Om19). Similarly, each cell of the control treatment received 1 mL of liquid MMN devoid of Om19. The microcuttings received intermittent mist for 15 s per 30 min under a light intensity of 100 μmol m^−2^ s^−1^. After three weeks in the propagation mist bed, the plug trays were moved into in a shaded greenhouse with a light level of 285 μmol m^−2^ s^−1^, temperature varying from 18 to 32 °C, and relative humidity ranging from 50% to 80%. Plants were fertigated with a solution containing Peters Professional 20–20–20 General Purpose Fertilizer (The Scotts Co., Marysville, OH, USA) with a N concentration of 100 mg L^−1^ and every other two weeks thereafter. Root initiation was monitored by gently digging a plug out of cell daily for a week. Root numbers were countered one month after transplanting by randomly taking three plugs from each tray. Number of Leaves, canopy height, and leaf area of the largest leaf were recorded after 30 and 60 days of growth in the plug trays. Plugs were then transplanted into 10-cm diameter plastic containers filled with the same Canadian peat-based substrate and top dressed with 3 g of a controlled-release fertilizer (Osmocote 19N-2.18P-7.47 K, 8–9 month, The Scotts Co., Marysville, OH, USA). Plants were grown in the same shaded greenhouse for additional three months. Number of leaves, canopy height, and leaf area of the largest leaf were recorded, and plant growth photographs were taken.

### Data analysis

Microcutting rooting data, fresh and dry weight, nutrient concentrations and total content, ex vitro growth parameters were subjected to analysis of variance (ANOVA) using SPSS Statistics (SPSS Inc., Chicago, IL, USA). If significance occurred, means were separated by Tukey’s HSD test at *P* < 0.05 or 0.01 level.

## Supplementary information

Table S1. Ex vitro growth data

Table S2. Supplementary Table 2
