# Supplementary material for: Ericoid mycorrhizal fungus enhances microcutting rooting of Rhododendron fortunei and subsequent growth
Source: Hortic Res. 2020 Sep 1;7:140. doi: 10.1038/s41438-020-00361-6 (PMC7459316; doi:10.1038/s41438-020-00361-6)
Supplement: Supplementary file 1 — Table S1. Ex vitro growth data [file 41438_2020_361_MOESM1_ESM.docx]

**Table S1. Rooting of** ***Rhododendron fortunei* microcuttings in a commercial Canadian peat-based substrate inoculated and uninoculated with Om19 and subsequent growth in a shaded greenhouse.**

| **Parameter** | **Day** | **30** | **60** | **150** |
| --- | --- | --- | --- | --- |
| Root No. | Uninoculated | 7.84 ± 1.25 | ND | ND |
|  | Inoculated | 10.56 ± 0.84* | ND | ND |
| Leaf No. | Uninoculated | 7.08 ± 1.02 | 9.17 ± 0.18 | 11.01 ± 0.68 |
|  | Inoculated | 8.14 ± 0.58 | 10.22 ± 1.33 | 13.62 ± 0.36* |
| Canopy height | Uninoculated | 18.33 ± 0.79 | 25.22 ± 1.37 | 31.13 ± 1.31 |
| (mm) | Inoculated | 25.83 ± 1.36* | 35.72 ± 1.40* | 59.13 ± 3.54** |
| Largest leaf | Uninoculated | 0.25 ± 0.03 | 0.45 ± 0.06 | 1.32 ± 0.26 |
| area (cm^2^) | Inoculated | 0.32 ± 0.15 | 1.25 ± 0.07** | 3.85 ± 0.43* |

Data were mean ± standard error (n = 3), where * and ** indicate significant differences in a given parameter between microcuttings inoculated and uninoculated with Om19 in each sampling date based on Tukey’s HSD test at *P* < 0.05 and *P* < 0.01 levels, respectively.

ND = not determined.
