# Supplementary material for: Ericoid mycorrhizal fungus enhances microcutting rooting of Rhododendron fortunei and subsequent growth
Source: Hortic Res. 2020 Sep 1;7:140. doi: 10.1038/s41438-020-00361-6 (PMC7459316; doi:10.1038/s41438-020-00361-6)
Supplement: Supplementary file 2 — Table S2. Supplementary Table 2 [file 41438_2020_361_MOESM2_ESM.docx]

**Table S2. RT-qPCR primers**

| Reference gene | Sequence |
| --- | --- |
| EF1a-F | TGTCATCGATGCTCCTGGAC |
| EF1a-R | TCTCGGGTCTGACCACCCTT |
|  |  |
|  |  |
| Selected Genes | Sequence |
| SymRK-F | AGTGGAGCTTGGTCGAATGG |
| SymRK-R | AATGCATCCTCCAGCTCTCG |
|  |  |
| DMI3-F | ACAGAACCAGGGAAATTGGATGAG |
| DMI3-R | GCGAAGGGAGGAGAGGACTAC |
|  |  |
| YUC3-F | CCCGGGCGGTGTATATTGAT |
| YUC3-R | GGCCCTTTCGAGGATGACAA |
|  |  |
| NRT-1-F | AGTGTTGCCAATGCCCTATTCTTC |
| NRT-1-R | ATATCGTTTGTCAGCCAGTTCGG |
|  |  |
| AMT-F | TCCTCCTCATTTCGTATATGTGGTAG |
| AMT-R | GCGTTGTCTCCTTTGTTCAACC |
|  |  |
| GS-1-F | GCATTGAGCAAGAGTACACCTTAC |
| GS-1-R | TGATTCCAGCATACAAGCAAGCC |
|  |  |
| GOGAT-1-F | CTTTTGATGGCGTCCTTGAG |
| GOGAT-1-R | TCCCACGGTTCCATAAGTGC |
|  |  |
| PHT-F | TGTCAATGGTGTCGCCCTTT |
| PHT-R | ACAGTTTTTGGCTCCCTCCC |
